# Apoptosis Induced by Tanshinone IIA and Cryptotanshinone Is Mediated by Distinct JAK/STAT3/5 and SHP1/2 Signaling in Chronic Myeloid Leukemia K562 Cells

**DOI:** 10.1155/2013/805639

**Published:** 2013-06-26

**Authors:** Ji Hoon Jung, Tae-Rin Kwon, Soo-Jin Jeong, Eun-Ok Kim, Eun Jung Sohn, Miyong Yun, Sung-Hoon Kim

**Affiliations:** ^1^College of Oriental Medicine, Kyung Hee University, 1 Hoegi-dong, Dongdaemun-gu, Seoul 130-701, Republic of Korea; ^2^Basic Herbal Medicine Research Group, Herbal Medicine Research Division, Korea Institute of Oriental Medicine, Daejeon 305-811, Republic of Korea

## Abstract

Though tanshinone IIA and cryptotanshinone possess a variety of biological effects such as anti-inflammatory, antioxidative, antimetabolic, and anticancer effects, the precise molecular targets or pathways responsible for anticancer activities of tanshinone IIA and cryptotanshinone in chronic myeloid leukemia (CML) still remain unclear. In the present study, we investigated the effect of tanshinone IIA and cryptotanshinone on the Janus activated kinase (JAK)/signal transducer and activator of transcription (STAT) signaling during apoptotic process. We found that both tanshinone IIA and cryptotanshinone induced apoptosis by activation of caspase-9/3 and Sub-G1 accumulation in K562 cells. However, they have the distinct JAK/STAT pathway, in which tanshinone IIA inhibits JAK2/STAT5 signaling, whereas cryptotanshinone targets the JAK2/STAT3. In addition, tanshinone IIA enhanced the expression of both SHP-1 and -2, while cryptotanshinone regulated the expression of only SHP-1. Both tanshinone IIA and cryptotanshinone attenuated the expression of bcl-x_L_, survivin, and cyclin D1. Furthermore, tanshinone IIA augmented synergy with imatinib, a CML chemotherapeutic drug, better than cryptotanshinone in K562 cells. Overall, our findings suggest that the anticancer activity of tanshinone IIA and cryptotanshinone is mediated by the distinct the JAK/STAT3/5 and SHP1/2 signaling, and tanshinone IIA has the potential for combination therapy with imatinib in K562 CML cells.

## 1. Introduction

*Salvia miltiorrhiza* Bunge (Danshen) is a traditional medicinal herb widely used for treating cardiovascular disease in Korea, China, and Japan [1]. To date, over 90 kinds of chemical constituents from *S. miltiorrhiza* have been reported [2, 3]. Of the phytochemicals, tanshinones are a group of lipophilic abietane diterpene compounds including tanshinone I, tanshinone IIA-B, cryptotanshinone, dihydrotanshinone I, isotanshinone I, and isocryptotanshinone I-II and have been extensively investigated [1, 4–7]. In particular, tanshinone IIA and cryptotanshinone have been presented the potential as anticancer drugs by targeting the multiple signaling pathways [8–18].

STAT family is transcriptional factors that play key roles in cytokine signaling [19]. STAT proteins are constitutively activated in cancer cells or tissues and thus have been suggested as attractive molecular target(s) for cancer therapy. In light of these events, numerous groups reported the inhibitory effects of plant polyphenols such as curcumin, resveratrol, piceatannol, and EGCG on STAT activation in various cancer cells [19, 20]. Tanshinone IIA and cryptotanshinone were also shown to have the inhibitory effects on the STAT activation in C6 glioma [21] and DU145 prostate cancer cells [22], respectively. However, there is no report on the molecular mechanisms leading to anticancer activity of tanshinone IIA and cryptotanshinone through the STAT signaling pathway in leukemia cells.

In the current study, we investigated the inhibitory effects of tanshinone IIA and cryptotanshinone on the activation of STAT3 or 5 linked to apoptosis in chronic myeloid leukemia (CML) K562 cells. Additionally, the synergistic effects of tanshinone IIA or cryptotanshinone with imatinib, a chemotherapeutic agent for CML, were examined by calculating combination index (CI).

## 2. Materials and Methods

### 2.1. Isolation of Tanshinone IIA and Cryptotanshinone

**Figure 1:**
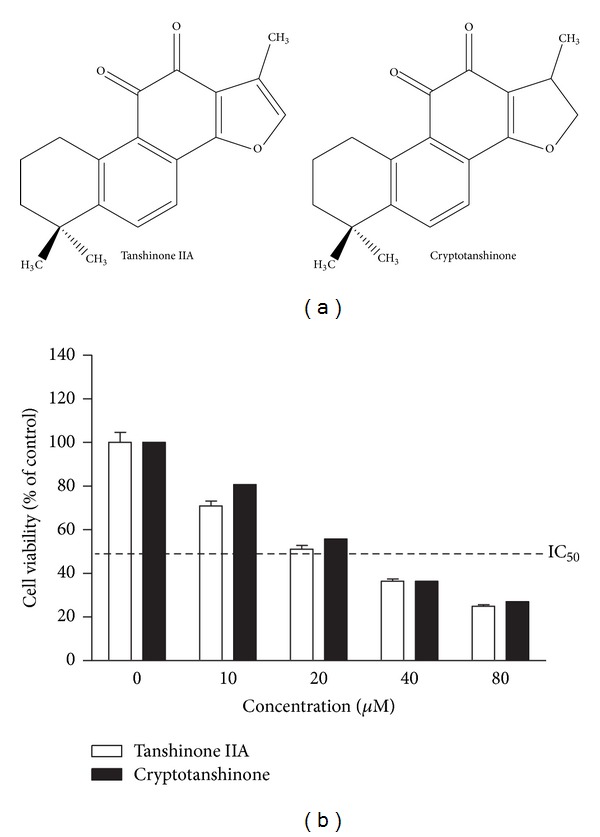
Tanshinone IIA and cryptotanshinone exert cytotoxicity in K562 cells. (a) Chemical structures of tanshinone IIA (left) and cryptotanshinone (right). (b) Cells were treated with various concentrations of tanshinone IIA or cryptotanshinone (0, 10, 20, 40, or 80 *μ*M) for 24 h. MTT assay was performed to evaluate the cytotoxicity.

Tanshinone IIA [23] and cryptotanshinone [24] (Figure 1(a)) were isolated as previously described. 

### 2.2. Cell Culture

Human chronic myeloid leukemia K562 cells were purchased from American Type Culture Collection (ATCC, Rockville, MD, USA) and maintained in RPMI 1640 medium supplemented 10% fetal bovine serum (FBS), 2 *μ*M L-glutamine, and penicillin/streptomycin.

### 2.3. Cytotoxicity Assay

Cytotoxic effects of tanshinone IIA or cryptotanshinone against K562 cells were evaluated by 3-(4,5-dimethylthiazol-2-yl)-2,5-diphenyltetrazolium bromide (MTT) assay. Cells were seeded onto 96-well microplates at a density of 2 × 10^4^ cells per well and exposed to various concentrations of tanshinone IIA or cryptotanshinone (0, 10, 20, 40, or 80 *μ*M) for 24 h. The cells were incubated with 3-(4,5-dimethylthiazol-2-yl)-2,5-diphenyl tetrazolium bromide (1 mg/mL) (Sigma Chemical Co., St. Louis, MO, USA) for 2 h and then with MTT lysis solution overnight. Optical density (OD) was measured using a microplate reader (Molecular Devices Co., Sunnyvale, CA, USA) at 570 nm. Cell viability was calculated as a percentage of viable cells in drug-treated group versus untreated control by the following equation:

(1) Cell  viability  (%) =[OD(Drug)−OD(Blank)][OD(Control)−OD(Blank)]×100.

### 2.4. Western Blot Analysis

K562 cells were lysed in lysis buffer (50 mM Tris-HCl, pH 7.4, 150 mM NaCl, 1% Triton X-100, 0.1% SDS, 1 mM EDTA, 1 mM Na_3_VO_4_, 1 mM NaF, and protease inhibitors cocktail). The extracts were incubated on ice for 30 min and supernatants were collected by centrifugation at 14,000 g at 4°C. The protein contents in the supernatant were measured by using a Bio-Rad DC protein assay kit II. Proteins were separated by electrophoresis on 12.5% SDS-PAGE gel and electrotransferred onto a Hybond ECL transfer membrane with transfer buffer (25 mM Tris, 250 mM glycine, and 20% methanol) at 300 mA for 90 min. The membrane was blocked in 5% nonfat skim milk and probed with primary antibodies for p-STAT3, p-STAT5, STAT3, STAT5, p-JAK2, JAK2 (Cell Signaling Tech., Danvers, MA, USA), SHP-1, SHP-2, bcl-x_L_, mcl-1, survivin, cyclin D1, cleaved caspase-9, cleaved caspase-3, poly(ADP-ribose) polymerase (PARP), and tubulin (Santa Cruz Biotechnologies, Santa Cruz, CA, USA), followed by incubating with horseradish peroxidase-(HRP-) conjugated secondary antibodies. Protein expression was detected by using enhanced chemiluminescence (ECL) system (Amersham Pharmacia, Piscataway, NJ, USA).

### 2.5. Electrophoretic Mobility Shift Assay (EMSA)

The STAT3 or STAT5/DNA binding activity was analyzed by EMSA using gel shift chemiluminescent EMSA kit (Active motif, Carlsbad, CA, USA). Nuclear extracts were incubated with STAT3 (5′-GAT CCT TCT GGG AAT TCC TAG ATC-3′) or STAT5 (5′-AGA TTT CTA ATT CAA TCC-3′) consensus oligonucleotides (Santa Cruz Biotechnology, Santa Cruz, CA, USA). The DNA/protein complex formed was separated from free oligonucleotides on 5% native polyacrylamide gels. Chemiluminescent detection was performed using ECL reagents according to the vendor's protocols (GE Health Care Bio-Sciences, Piscataway, NJ, USA).

### 2.6. Cell Cycle Analysis

Cell cycle analysis was performed by PI staining. K562 cells were treated with tanshinone IIA or cryptotanshinone for 24 h, collected and fixed in 70% ethanol. The cells were then incubated at 37°C with 0.1% RNase A in PBS for 30 min and suspended in PBS containing 25 *μ*g/mL PI for 30 min at room temperature. The stained cells were analyzed for DNA content in FACSCalibur (Becton Dickinson, Franklin Lakes, NJ, USA) using the Cell Quest program (Becton Dickinson, Franklin Lakes, NJ, USA).

### 2.7. Apoptosis Analysis by Annexin V-PI Double Staining

Apoptosis of the cryptotanshinone or tanshinone IIA-treated cells was quantitated by double staining with Annexin V-FITC and PI using the Annexin V-Apoptosis Detection kit (Biovision, Milpitas, CA, USA) according to the manufacturer's instructions. Apoptotic cells were analyzed by FACSCalibur (Becton Dickinson, San Jose, CA, USA) to be defined as those positive for Annexin V with or without PI staining.

### 2.8. Propidium Iodide (PI) Staining

K562 cells were exposed to tanshinone IIA or cryptotanshinone and plated onto poly-L-lysine-coated slide glass. The cells were fixed in 70% ethanol and stained with PI solution (50 *μ*g/mL) (BD Biosciences, Bedford, MA, USA) containing 100 *μ*g/mL RNase for 10 min. The slides were mounted with 70% glycerol in PBS and visualized under an Axio vision 4.0 fluorescence microscope (Carl Zeiss Inc., Weimar, Germany).

### 2.9. Combination Index (CI) Calculation

The CI was determined by Chou-Talalay method and CalcuSyn software (Biosoft, Ferguson, MO, USA). A CI of less than 1 was considered synergistic [25].

### 2.10. Statistical Analyses

All data were presented as means ± standard deviation (SD). Statistical significance was verified by Student's *t*-test using SigmaPlot software (Systat Software Inc., San Jose, CA, USA).

## 3. Results

### 3.1. Tanshinone IIA and Cryptotanshinone Exert Cytotoxicity against Chronic Myeloid Leukemia K562 Cells

To compare the cytotoxicity of tanshinone IIA and cryptotanshinone in K562 cells, MTT assay was performed. Cells were treated with various concentrations (0, 10, 20, 40, or 80 *μ*M) for 24 h. Both tanshinone IIA and cryptotanshinone substantially reduced the cell viability in a dose-dependent manner (Figure 1(b)). There was no significant difference in the cytotoxicity between two chemicals in the cells (IC_50_ = ~20 *μ*M).

### 3.2. Tanshinone IIA Inhibits STAT5, but Not STAT3, Signaling in K562 Cells

**Figure 2:**
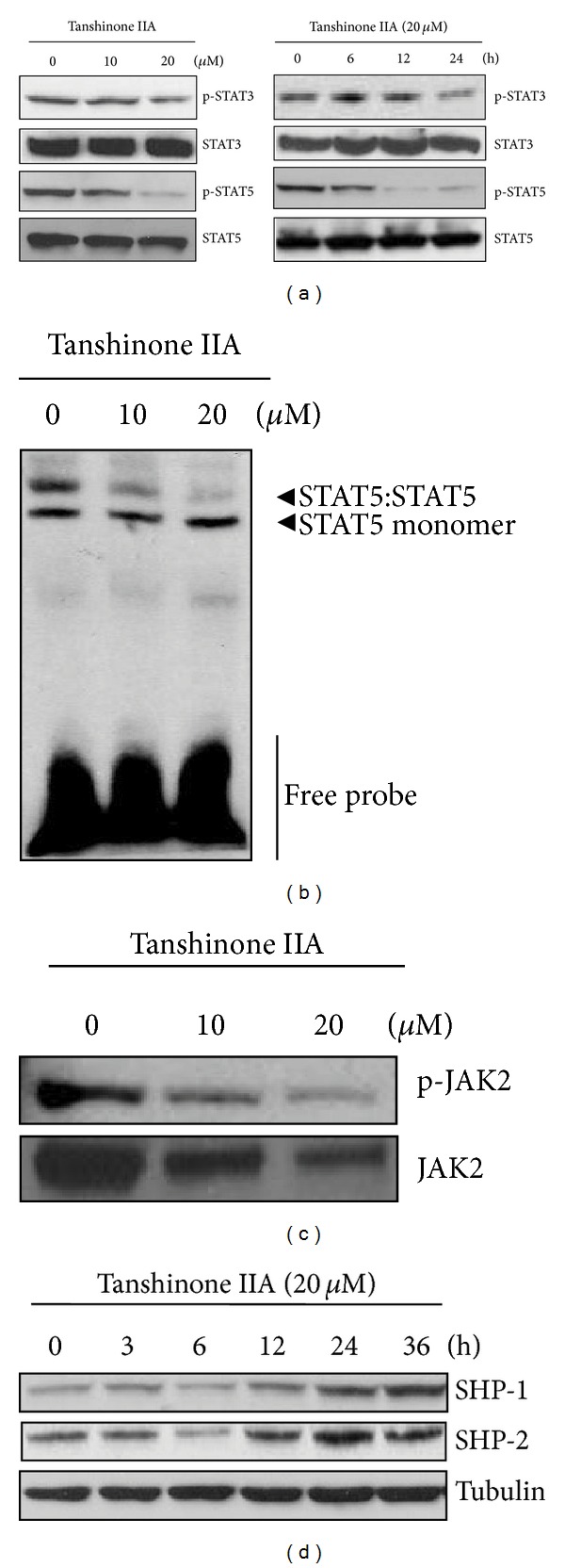
Tanshinone IIA inactivates STAT5, but not STAT3, in K562 cells. (a) Cells were treated with tanshinone IIA (0, 10, or 20 *μ*M) for 24 h (left) or 20 *μ*M for 0, 6, 12, or 24 h (right). Cell lysates were prepared and subjected to Western blotting for phospho-STAT3 and phospho-STAT5. (b) Cells were treated with tanshinone IIA (0, 10, or, 20 *μ*M) for 24 h. Gel shift mobility assay was performed to determine the STAT5/DNA binding activity. (c) Cells were treated with tanshinone IIA (0, 10, or 20 *μ*M) for 24 h. Western blotting was performed to detect phosphorylation of JAK2. (d) Cells were treated with 20 *μ*M tanshinone IIA for 0, 3, 6, 12, 24, or 36 h. Western blotting was conducted to determine the expression of SHP-1 and SHP-2.

Effects of tanshinone IIA on STAT3 and 5 activation were examined by Western blot analysis. As shown in Figure 2(a), tanshinone IIA treatment significantly inhibited the phosphorylation of STAT5, but not STAT3, in a dose- and time-dependent manner. We further confirmed the inhibitory effect of tanshinone IIA on STAT5 by gel shift mobility assay. Consistent with the results of immunoblotting, tanshinone IIA prevented the STAT5/DNA binding in a dose-dependent manner (Figure 2(b)). To find out whether tyrosine kinases mediate the tanshinone IIA-initiated STAT5 inactivation, the effects of tanshinone IIA on the phosphorylation of JAK1, 2 and c-Src in K562 cells were examined. The results revealed that tanshinone IIA led to dephosphorylation of JAK2 (Figure 2(c)), but not JAK1 and c-Src (data not shown). Furthermore, we observed that tanshinone IIA enhanced expression of tyrosine phosphatase SHP-1 and -2 in a time-dependent manner (Figure 2(d)).

### 3.3. Cryptotanshinone Inhibits STAT3, but Not STAT5, Signaling in K562 Cells

**Figure 3:**
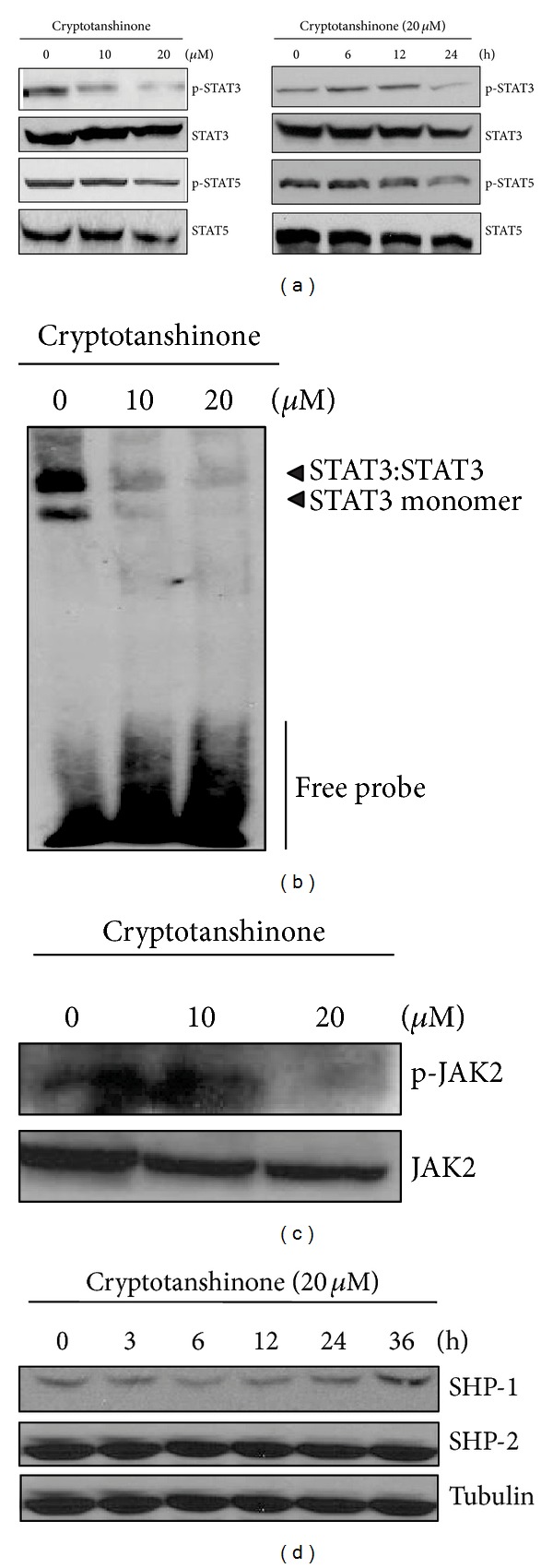
Cryptotanshinone inactivates STAT3, but not STAT5, in K562 cells. (a) Cells were treated with cryptotanshinone (0, 10, or 20 *μ*M) for 24 h (left) or 20 *μ*M for 0, 6, 12, or 24 h (right). Cell lysates were prepared and subjected to Western blotting for phospho-STAT3 and phospho-STAT5. (b) Cells were treated with cryptotanshinone (0, 10, or 20 *μ*M) for 24 h. Gel shift mobility assay was performed to determine the STAT3/DNA binding activity. (c) Cells were treated with cryptotanshinone (0, 10, or 20 *μ*M) for 24 h. Western blotting was performed to detect phosphorylation of JAK2. (d) Cells were treated with 20 *μ*M cryptotanshinone for 0, 3, 6, 12, 24, or 36 h. Western blotting was conducted to determine the expression of SHP-1 and SHP-2.

Parallel assays were carried out in cryptotanshinone-treated K562 cells. Different from tanshinone IIA, cryptotanshinone reduced the phosphorylation level of STAT3, but not STAT5, in a dose- and time-dependent manner (Figure 3(a)). In addition, cryptotanshinone suppressed the binding of STAT3 to DNA in a dose-dependent manner (Figure 3(b)). However, cryptotanshinone also inhibited the phosphorylation of JAK2, an upstream kinase of STAT3 or 5 in the cells (Figure 3(c)). Besides, cryptotanshinone led to increased expression of SHP-1, but no effect on the expression of SHP-2 (Figure 3(d)).

### 3.4. Tanshinone IIA and Cryptotanshinone Induce Apoptosis in K562 Cells

**Figure 4:**
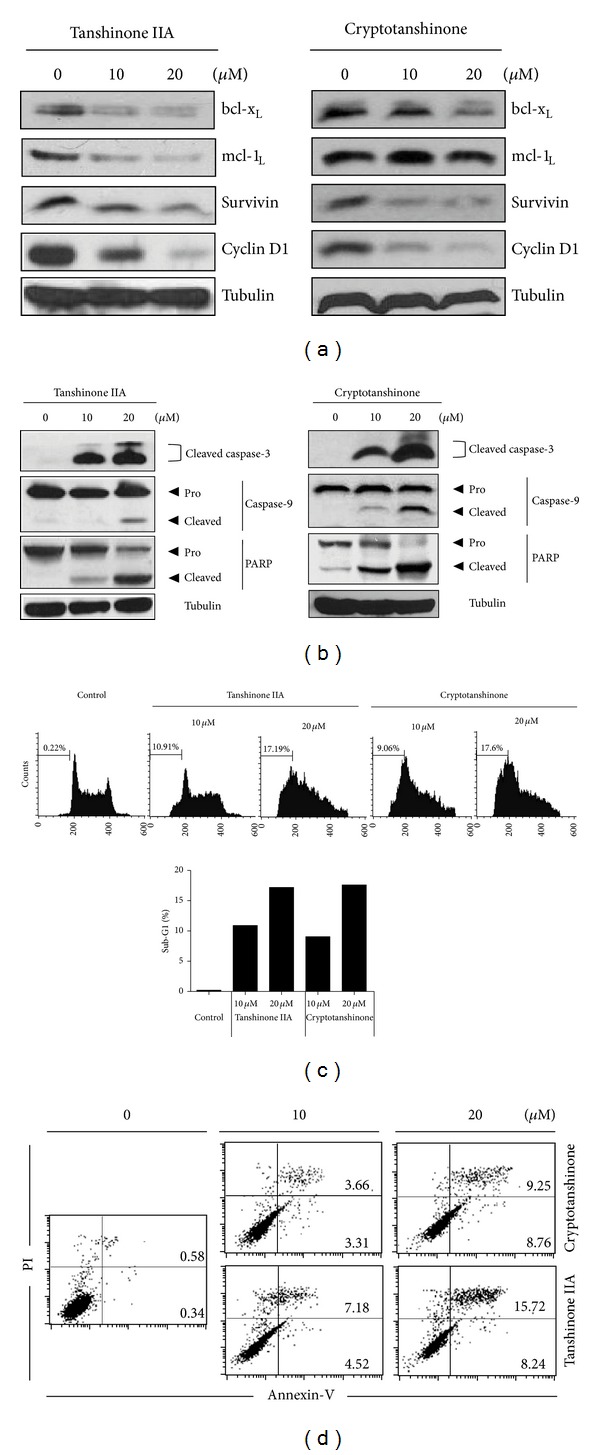
Tanshinone IIA and cryptotanshinone induce apoptosis in K562 cells. (a and b) Cells were treated with tanshinone IIA (left) or cryptotanshinone (right) for 24 h. Cell lysates were prepared and subjected to Western blotting to detect expression of apoptosis-related proteins bcl-x_L_, mcl-1_L_, surviving, and cyclin D1 (a) and caspase-3, caspase-9, and PARP (b). (c) Flow cytometry analysis of sub-G1 apoptotic DNA fraction of the cells treated with tanshinone IIA or cryptotanshinone (0, 10, or 20 *μ*M) for 24 h. After fixation in 75% ethanol, cells were stained with PI and analyzed by flow cytometry. (d) Cells were treated with tanshinone IIA or cryptotanshinone (10 or 20 *μ*M) for 24 h. Percentage of apoptotic cells in tanshinone IIA or cryptotanshinone-treated cells by Annexin V-PI staining. Each experiment was repeated three times.

JAK/STAT signaling regulates gene products involved in various cellular processes such as survival, proliferation, and cell cycle progression [20, 26, 27]. Both tanshinone IIA and cryptotanshinone significantly attenuated the expression of STAT-related survival genes such as bcl-x_L_, surviving, and cyclin D1 in a dose-dependent manner (Figure 4(a)). However, only tanshinone IIA, but not cryptotanshinone, suppressed the expression of antiapoptotic mcl-1_L_ in K562 cells (Figure 4(a), left panel). To confirm that tanshinone IIA or cryptotanshinone can induce apoptosis, activation of caspase-9 and -3, key molecules in intrinsic apoptosis pathway [28], was evaluated by immunoblotting. As expected, both tanshinone IIA and cryptotanshinone clearly induced the cleavages of caspase-9 and -3 as well as PARP in a dose-dependent manner (Figure 4(b)). Consistently, cell cycle analysis showed increased accumulation of the sub-G1 cell from 0.22% to 17.19% or 17.60% by tanshinone IIA or cryptotanshinone in K562 cells, respectively (Figure 4(c)). Moreover, we found that treatment of 20 *μ*M tanshinone IIA or cryptotanshinone dramatically increased the apoptotic cell population by Annexin V-PI double staining to 23.96 and 18.01%, respectively (Figure 4(d)).

### 3.5. Tanshinone IIA and Cryptotanshinone Synergistically Promote Anticancer Effects with Imatinib in K562 Cells

**Figure 5:**
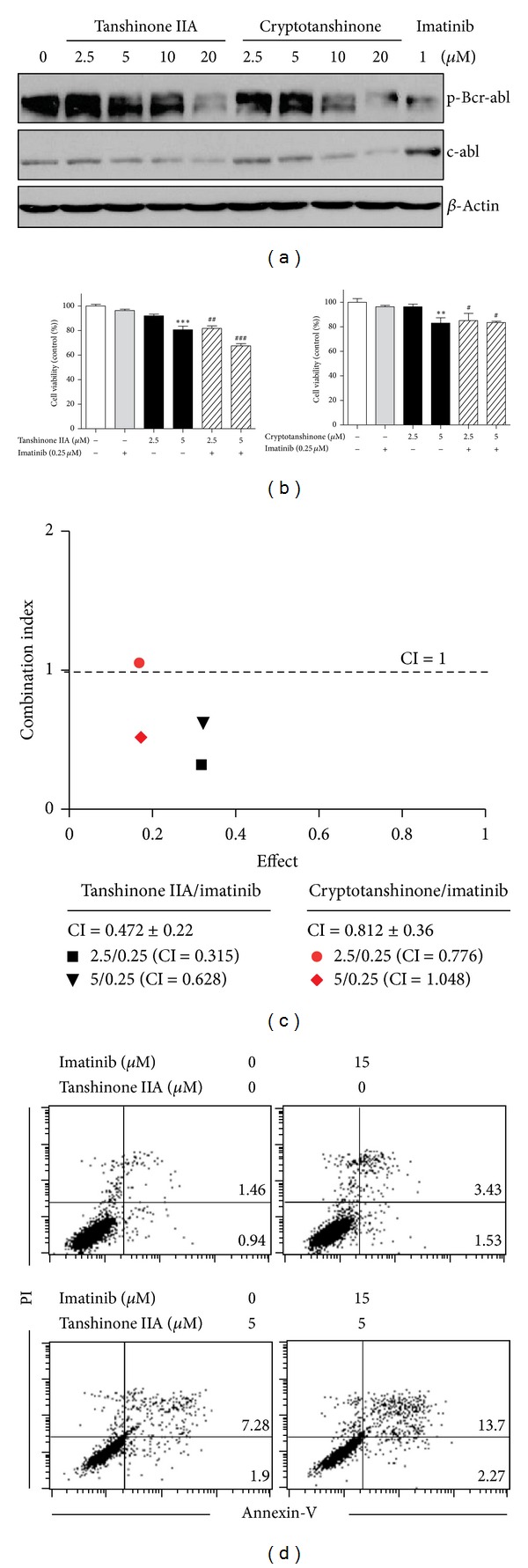
Tanshinone IIA and cryptotanshinone with imatinib synergistically inhibit the viability of K562 cells. (a) Cells were treated with various concentrations of tanshinone IIA or cryptotanshinone (0, 2.5, 5, 10, or 20 *μ*M) for 24 h. Cell lysates were prepared and subjected to Western blotting for phospho-bcr-abl. (b) Cells were treated with tanshinone IIA (left) or cryptotanshinone (right) and/or imatinib for 24 h. Cell viability was measured by MTT assay. (c) The combination index (CI) between two drugs was determined by Chou-Talalay method and CalcuSyn software (Biosoft, Ferguson, MO, USA). (d) Cells were treated with tanshinone IIA and imatinib for 24 h at the same time. Percentage of apoptotic cells in cotreated cells by Annexin V-PI double staining.

Bcr-abl is an abnormal gene formed by the reciprocal translocation between chromosomes 9 and 22 in CML [29]. We examined whether tanshinone IIA or cryptotanshinone can affect activation of bcr-abl by Western blotting. As shown in Figure 5(a), both tanshinone IIA and cryptotanshinone reduced phosphorylation of bcr-abl in a dose-dependent manner. Then, to test the synergy between tanshinone IIA or cryptotanshinone and imatinib, a competitive tyrosine kinase inhibitor used in the treatment of CML [30], K562 cells were cotreated with tanshinone IIA or cryptotanshinone (0, 2.5, or 5 *μ*M) in the absence or presence of imatinib (0.25 *μ*M) for 24 h. The cell viability was significantly decreased in combination of tanshinone IIA or cryptotanshinone with imatinib in a dose-dependent manner compared to untreated control (Figure 5(b)). Tanshinone IIA remarkably showed the synergistic effect on the imatinib-induced apoptosis with CI value = 0.315 and 0.628 at 2.5 and 5 *μ*M, respectively (Figure 5(c)). In contrast, cryptotanshinone treatment with imatinib had the synergistic effect only at 2.5 *μ*M (CI = 0.776) while showing the additional effect at 5 *μ*M (CI = 1.048) (Figure 5(c)). Furthermore, combination treatment of imatinib and tanshinone IIA synergistically increased the apoptotic population of Annexin V-PI double positive stained cells to 16%, while single treatment of imatinib or tanshinone IIA induced 4.96% and 9.18% apoptosis in K562, respectively (Figure 5(d)).

## 4. Discussion

Phytochemicals are natural compounds in plants such as fruits, vegetables, beans, grains, and others. In the American Cancer Society (ACS) report in 2008, some phytochemicals may account for the beneficial effects in humans to prevent and treat many health conditions. For this reason, these phytochemicals have been thought as valuable materials to develop new therapeutic drug or dietary supplement. Recently, numerous papers have reported the potential of phytochemicals to ameliorate the various diseases such as cancer, inflammation, metabolic syndrome, and cardiovascular disease. In the present study, we comparatively investigated the anticancer mechanism of tanshinone IIA and cryptotanshinone from *S. miltiorrhiza* in CML, a form of leukemia characterized by the increased and unregulated growth of predominantly myeloid cells in the bone marrow [31]. Our group recently reported that tanshinone IIA induces apoptosis through activation of c-jun N-terminal kinase in KBM-5 cells [18]. Ge et al. reported that cryptotanshinone mediates cell cycle arrest and apoptosis of multidrug-resistant K562/ADM cells by inactivating eukaryotic initiation factor 4E [10]. Additionally, we also reported that cryptotanshinone enhances TNF-*α*-induced apoptosis in KBM-5 cells [11]. Nonetheless, the molecular mechanisms leading to anti-CML properties of tanshinone IIA and cryptotanshinone are not fully understood yet.

STAT is one of the important transcriptional factor families and plays crucial roles as a molecular target for cancer prevention and therapy [32]. STAT family consists of 7 different subfamilies STAT1, 2, 3, 4, 5a, 5b, and 6, and STAT3 and 5 are constitutively activated in cancer cells. STAT 3 and 5 are activated by nonreceptor tyrosine kinases of the Janus family (JAK) and c-Src [33, 34], and protein tyrosine phosphatases (PTPs) such as Src homology 2 domain-containing phosphatases (SHPs), phosphatase and tensin homolog (PTEN), and suppressor of cytokine signaling proteins (SOCS) are also linked to STAT signaling [35]. Therefore, the JAK/STAT3 or 5 signaling has been thought as a valuable molecular target for cancer therapy [34, 36].

**Figure 6:**
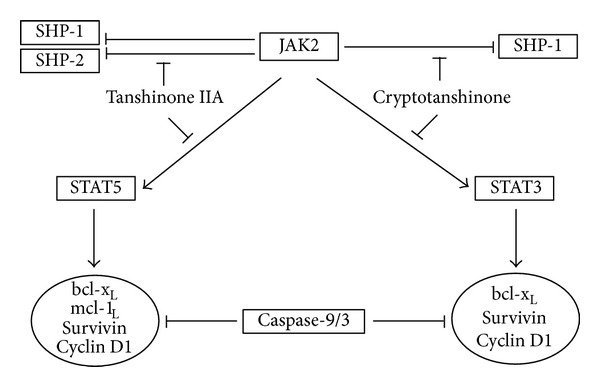
Schematic diagram indicating the effect of tanshinone IIA and cryptotanshinone on JAK/STAT signaling and apoptosis pathways in CML cells.

In our study, we found that both tanshinone IIA and cryptotanshinone reduced the phosphorylation of JAK2, an upstream kinase of STATs, in K562 CML cells. However, the effects of tanshinone IIA and cryptotanshinone on STAT activation were clearly different in K562 cells. Tanshinone IIA reduced the phosphorylation of STAT5, but not STAT3, and consistently prevented the STAT5/DNA binding in the cells. In contrast, cryptotanshinone inactivated STAT3, but not STAT5, at posttranslational and transcriptional levels. In addition, tanshinone IIA induced the expression of SHP-1 and -2 whereas cryptotanshinone increased the expression of SHP-1, but not SHP-2, in K562 cells (Figure 6).

The JAK/STAT signaling is involved in oncogenesis and cancer progression through upregulation of antiapoptotic genes [37]. Tanshinone IIA and cryptotanshinone commonly repressed the expression of bcl-x_L_, survivin and cyclin D1 in K562 cells. In contrast, only tanshinone IIA, but not cryptotanshinone, decreased the mcl-1_L_ expression. Apoptosis induction by tanshinone IIA or cryptotanshinone was confirmed by activation of caspase-9 and -3, cell cycle analysis and nuclear staining using PI (Figure 6). Although tanshinone IIA and cryptotanshinone exerted anti-CML activities in a different way by targeting the distinct STAT signaling, there was no significant difference in the induction of apoptosis by them. Further studies are necessary such as gene silencing for SHP-2 or mcl-1_L_ to verify the precise mechanisms responsible for the different regulation between tanshinone IIA and cryptotanshinone against CML cells in the near future. Using stable cells overexpressed STAT3 or 5 will be also beneficial tools to prove the anti-CML mechanisms.

Bcr-Abl selective tyrosine kinase inhibitor, imatinib (marketed by Novartis as Gleevec), has been extensively used for CML therapy [30]. However, despite of its specific therapeutic effect for CML, serious adverse effects and cost problem can limit the use of imatinib. In the current study, we tested the possibility that tanshinone IIA or cryptotanshinone can stimulate anti-CML effect induced by imatinib by lowering dosage in K562 cells. Our data revealed that tanshinone IIA enhanced imatinib-induced cell death more effectively than cryptotanshinone, with CI value <1 even at 2.5 *μ*M, determined by Chou-Talalay method and CalcuSyn software, implying significant synergy between tanshinone IIA and imatinib as a potent combination therapy for CML. However, additional experiments are required using *in vivo* mouse xenograft model to validate the *in vitro* studies.

In summary, tanshinone IIA inhibited JAK2/STAT5 signaling, whereas cryptotanshinone targets the JAK2/STAT3 in K562 cells. Furthermore, tanshinone IIA enhanced the expression of both SHP-1 and -2, while cryptotanshinone regulated the expression of only SHP-1. Also, both tanshinone IIA and cryptotanshinone attenuated the expression of STAT-related genes such as bcl-x_L_, survivin, and cyclin D1.

## 5. Conclusion

Our findings clearly demonstrate that anticancer activity of tanshinone IIA and cryptotanshinone is mediated by the distinct JAK/STAT3/5 and SHP1/2 signaling in K562 cells. Of note, tanshinone IIA showed more potential for the synergy with imatinib compared with cryptotanshinone as a potent candidate for combination therapy.
