# Corrigendum to “Apoptosis Induced by Tanshinone IIA and Cryptotanshinone Is Mediated by Distinct JAK/STAT3/5 and SHP1/2 Signaling in Chronic Myeloid Leukemia K562 Cells”

**DOI:** 10.1155/2018/1295359

**Published:** 2018-12-02

**Authors:** Ji Hoon Jung, Tae-Rin Kwon, Soo-Jin Jeong, Eun-Ok Kim, Eun Jung Sohn, Miyong Yun, Sung-Hoon Kim

**Affiliations:** ^1^College of Oriental Medicine, Kyung Hee University, 1 Hoegi-dong, Dongdaemun-gu, Seoul 130-701, Republic of Korea; ^2^Basic Herbal Medicine Research Group, Herbal Medicine Research Division, Korea Institute of Oriental Medicine, Daejeon 305-811, Republic of Korea

In the article titled “Apoptosis Induced by Tanshinone IIA and Cryptotanshinone Is Mediated by Distinct JAK/STAT3/5 and SHP1/2 Signaling in Chronic Myeloid Leukemia K562 Cells” [1], there was an error in Figure 3(d), where the blots for SHP-2 and Tubulin were duplicated by mistake. Some of the underlying blots are available in the Supplementary Materials (available here). The figure should be corrected as below.

## Supplementary Materials

Supplementary Materials Original Western blot images.

## Figures and Tables

**Figure 3:**
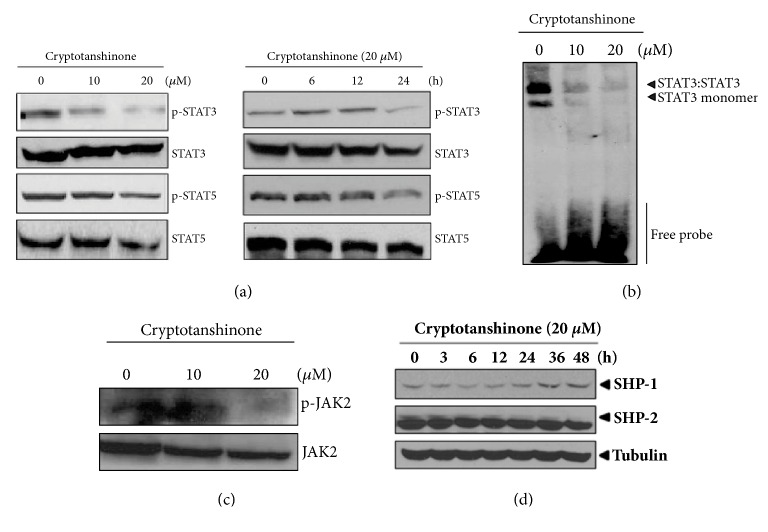
Cryptotanshinone inactivates STAT3, but not STAT5, in K562 cells. (a) Cells were treated with cryptotanshinone (0, 10, or 20 *μ*M) for 24 h (left) or 20 *μ*M for 0, 6, 12, or 24 h (right). Cell lysates were prepared and subjected to Western blotting for phospho-STAT3 and phospho-STAT5. (b) Cells were treated with cryptotanshinone (0, 10, or 20 *μ*M) for 24 h. Gel shift mobility assay was performed to determine the STAT3/DNA binding activity. (c) Cells were treated with cryptotanshinone (0, 10, or 20 *μ*M) for 24 h. Western blotting was performed to detect phosphorylation of JAK2. (d) Cells were treated with 20 *μ*M cryptotanshinone for 0, 3, 6, 12, 24, or 36 h. Western blotting was conducted to determine the expression of SHP-1 and SHP-2.
