# Supplementary figures and images for: Corrigendum to “Apoptosis Induced by Tanshinone IIA and Cryptotanshinone Is Mediated by Distinct JAK/STAT3/5 and SHP1/2 Signaling in Chronic Myeloid Leukemia K562 Cells”
Source: Evid Based Complement Alternat Med. 2018 Dec 2;2018:1295359. doi: 10.1155/2018/1295359 (PMC6304535; doi:10.1155/2018/1295359)

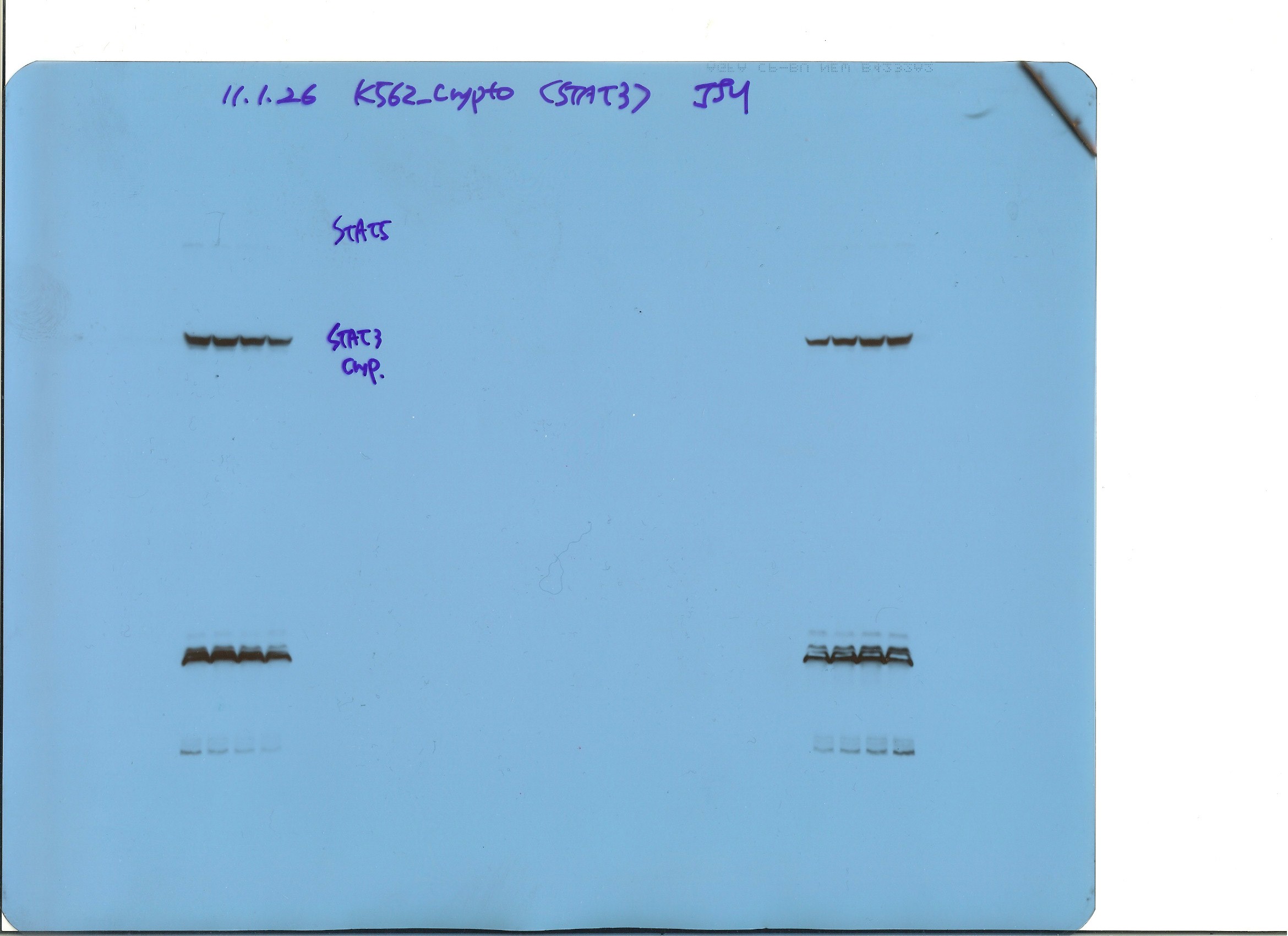

Supplement: Supplementary Materials — Original Western blot images. [file 1295359.f1.zip › 805639.Raw Data.0.jpg]

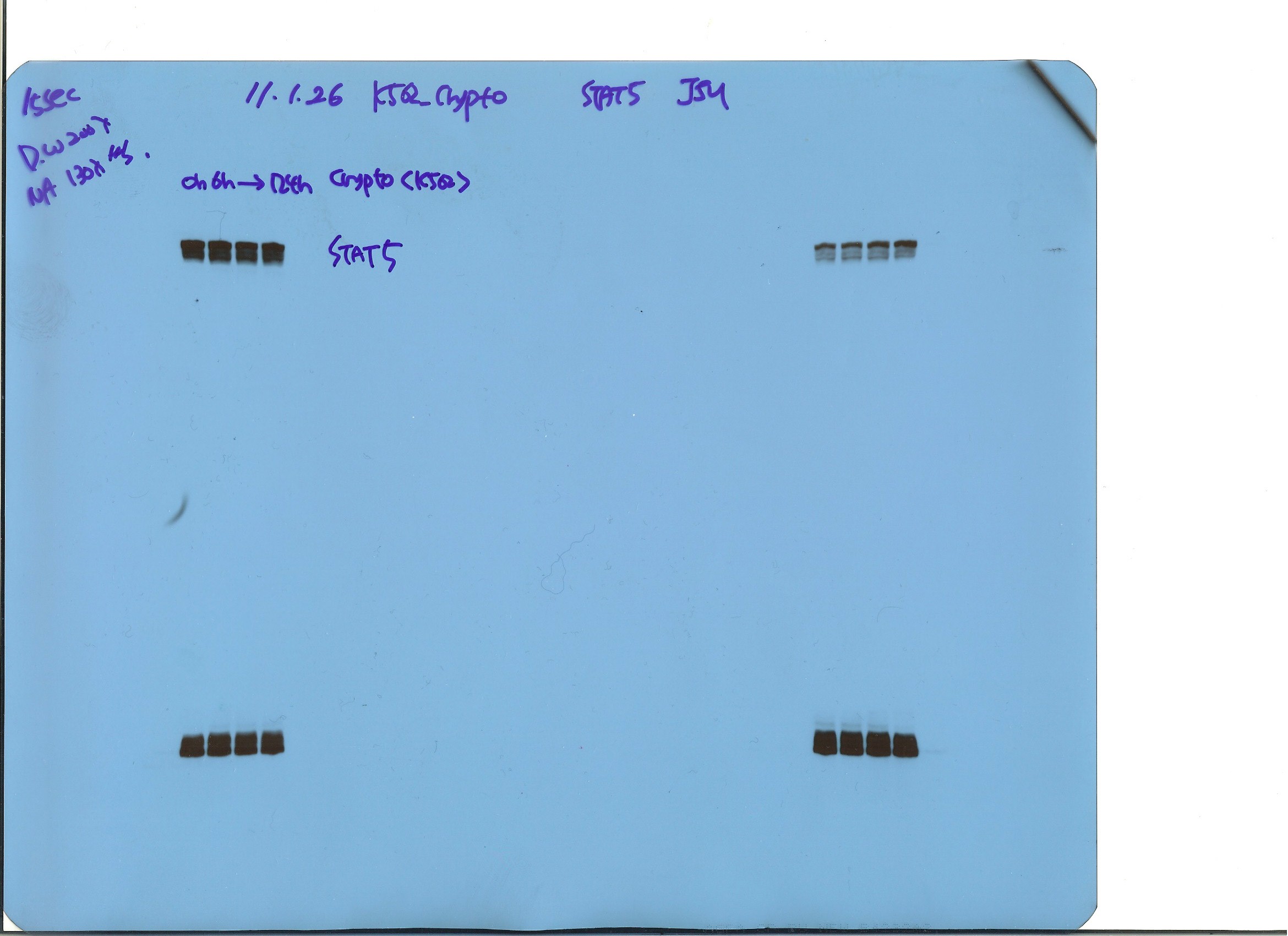

Supplement: Supplementary Materials — Original Western blot images. [file 1295359.f1.zip › 805639.Raw Data.1.jpg]

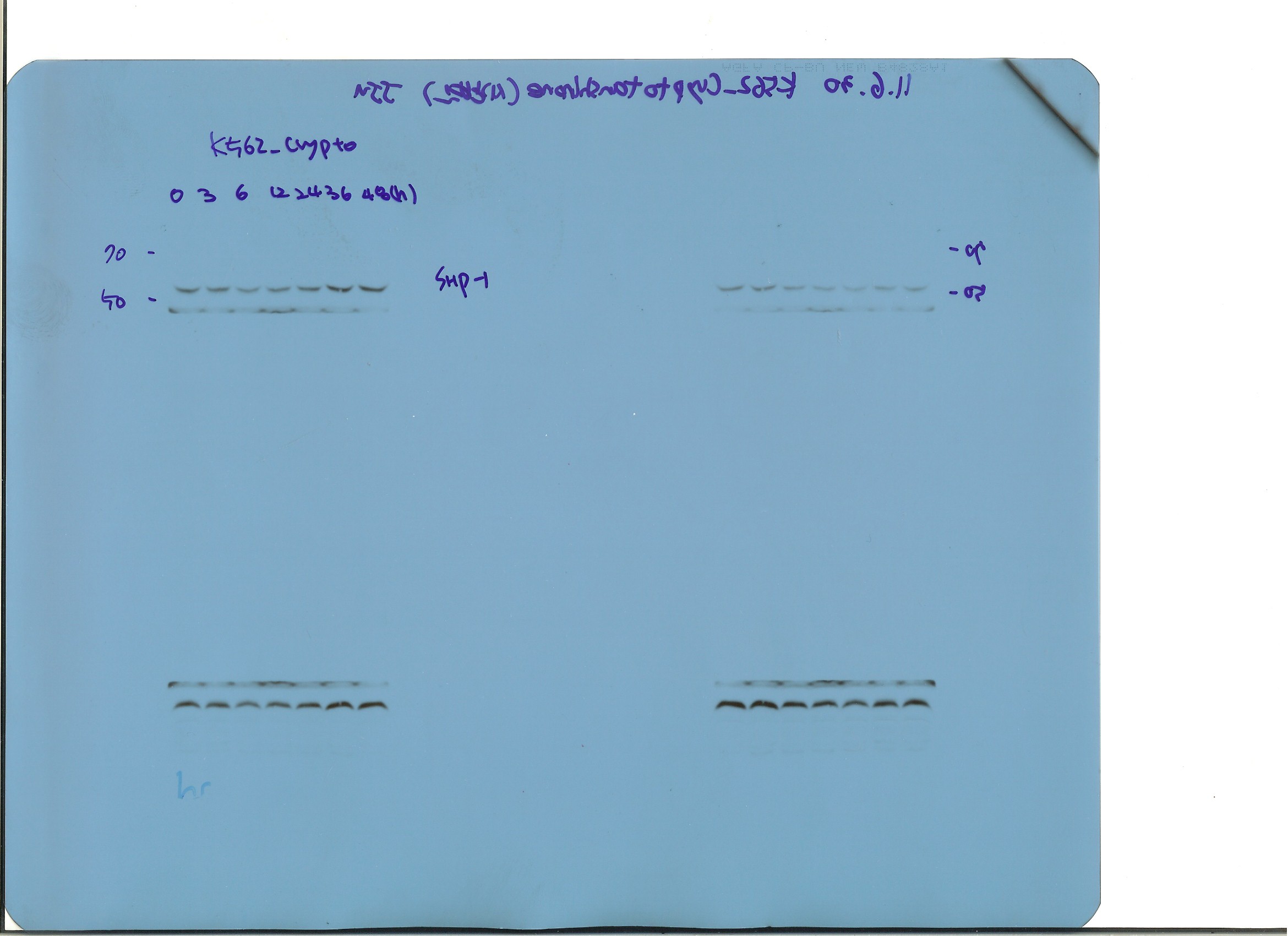

Supplement: Supplementary Materials — Original Western blot images. [file 1295359.f1.zip › 805639.Raw Data.2.jpg]

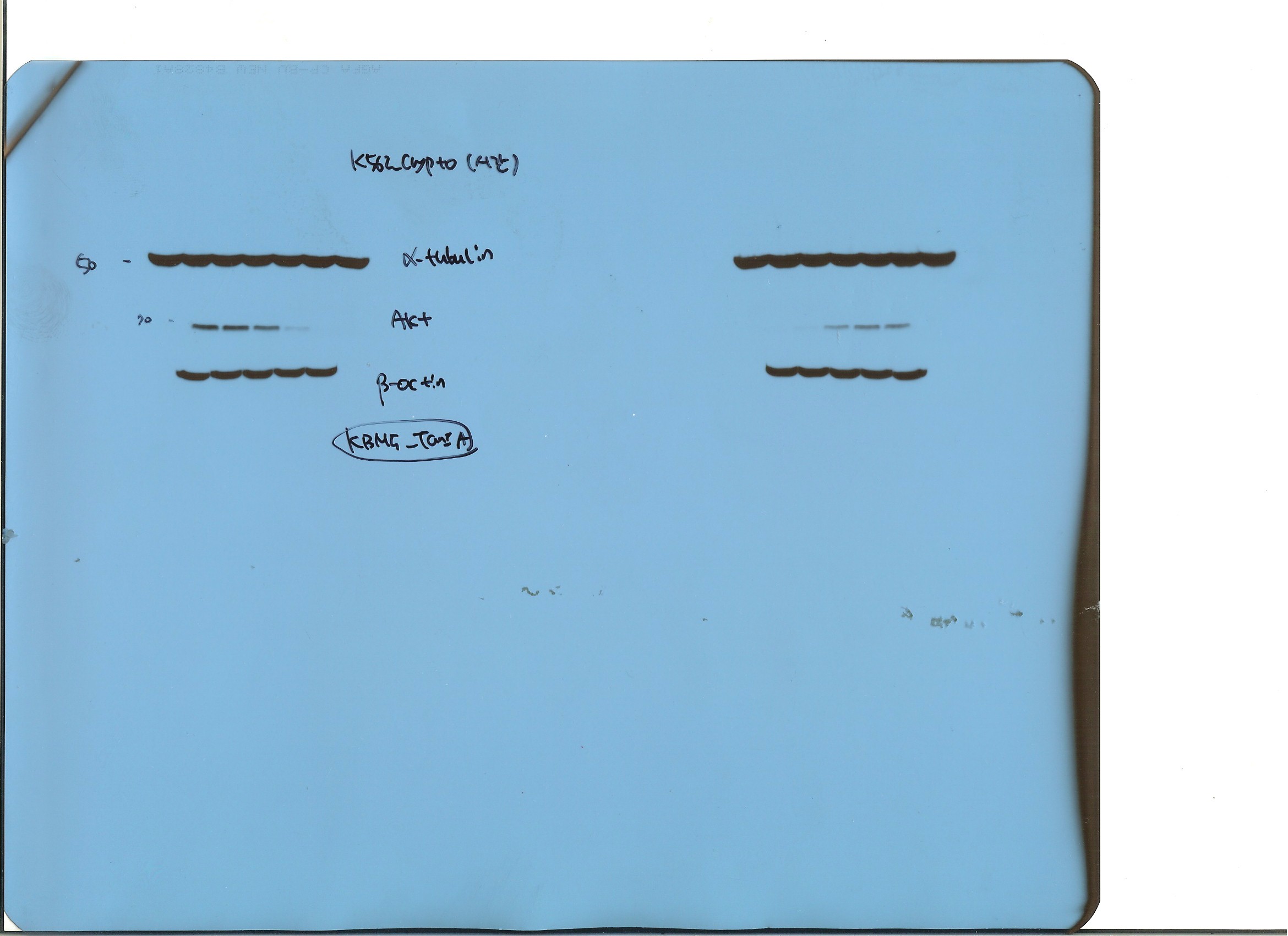

Supplement: Supplementary Materials — Original Western blot images. [file 1295359.f1.zip › 805639.Raw Data.3.jpg]

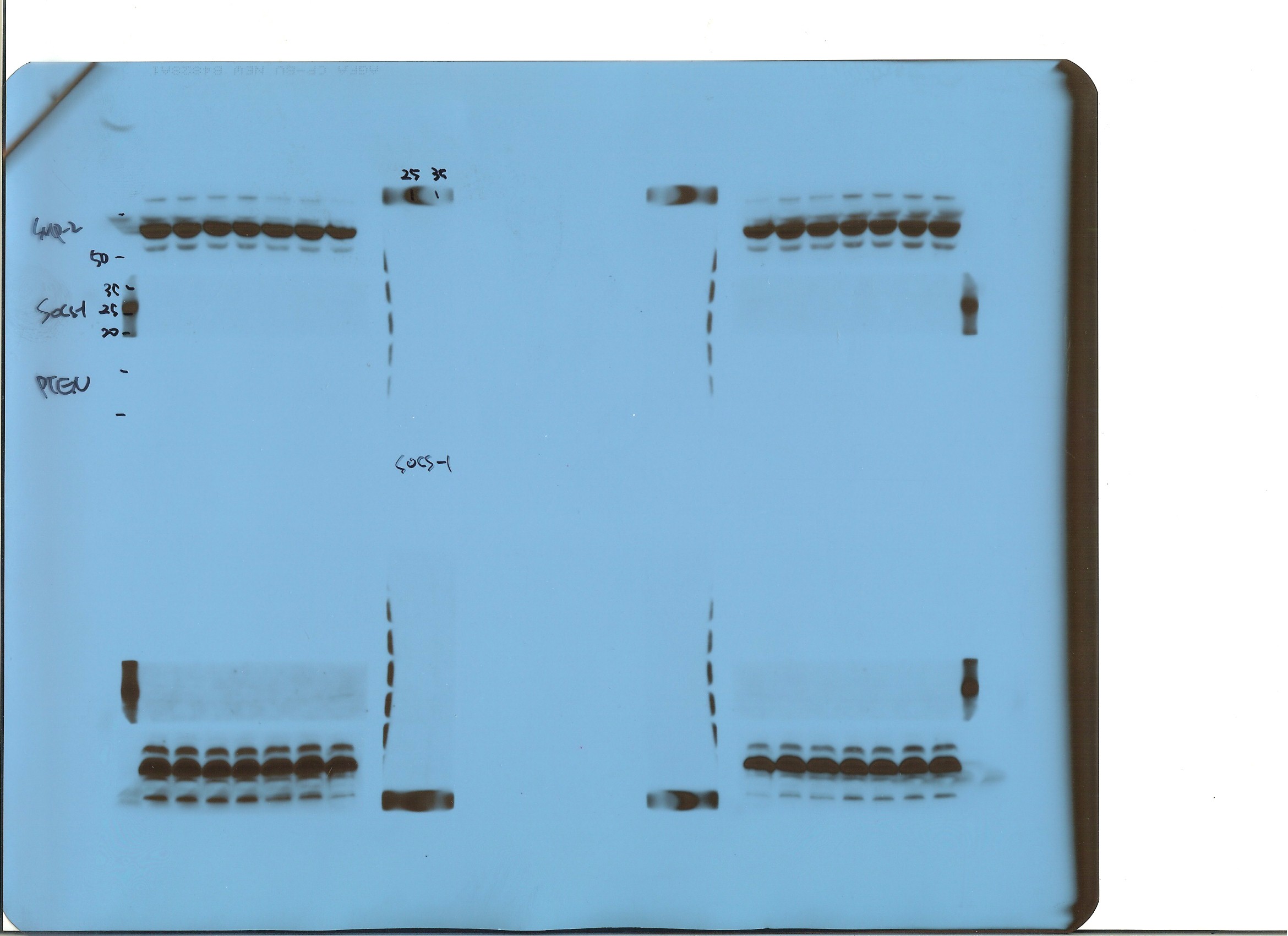

Supplement: Supplementary Materials — Original Western blot images. [file 1295359.f1.zip › 805639.Raw Data.4..jpg]

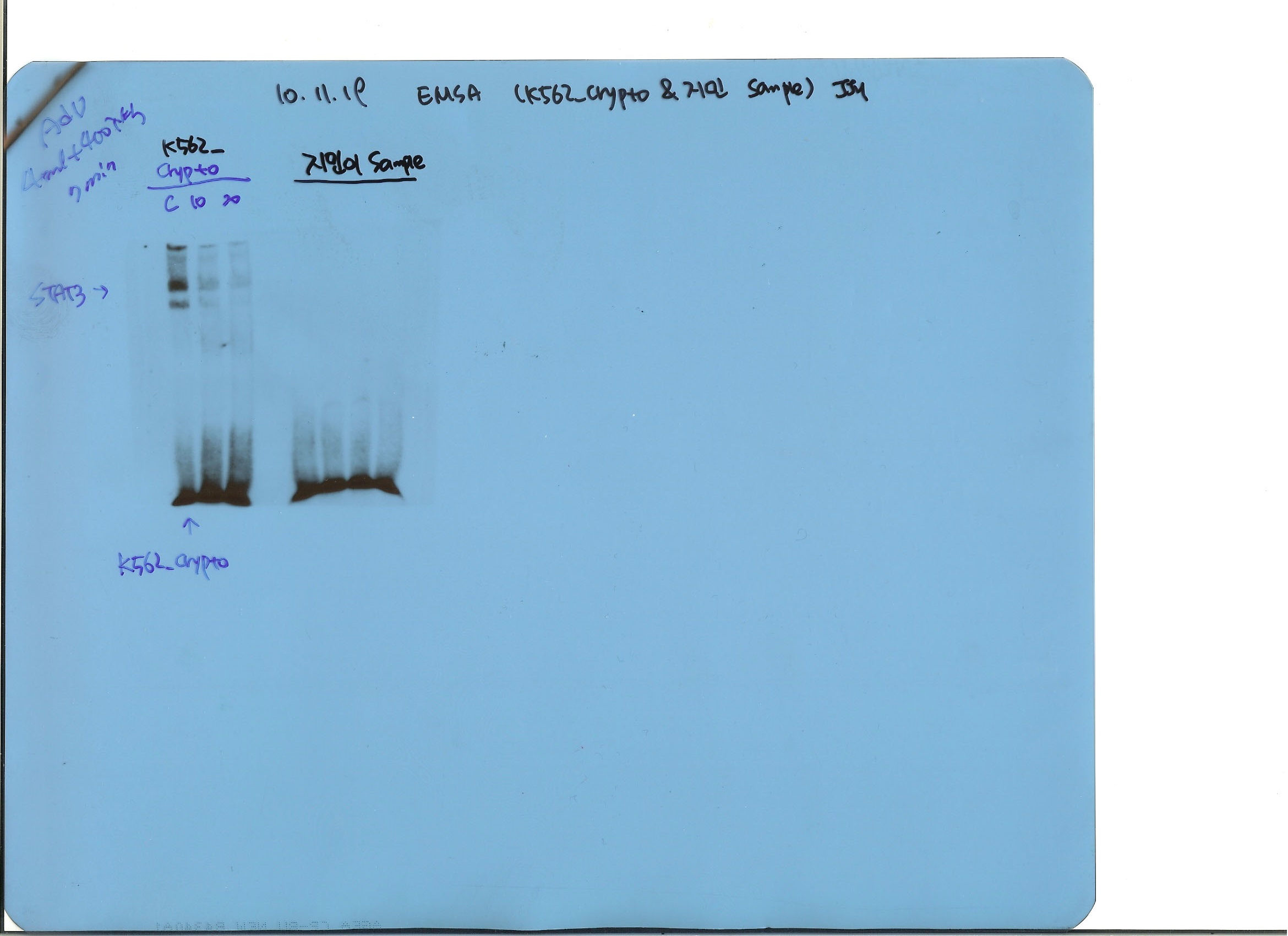

Supplement: Supplementary Materials — Original Western blot images. [file 1295359.f1.zip › 805639.Raw Data.5.jpg]
